# Comparative Study of Nebulised Dexmedetomidine Versus Nebulised Clonidine for Postoperative Sore Throat in Patients Undergoing Elective Laparoscopic Cholecystectomy

**DOI:** 10.7759/cureus.93802

**Published:** 2025-10-04

**Authors:** Vandana Mehra, Ashutosh Kaushal, Sri Rama Ananta Nagabhushanam Padala, Anuj Jain, Rudrashish Haldar, Pfokreni L, Ujjwal Gupta, Rhea Thotungal, Surya Kant, Sweta Kumari

**Affiliations:** 1 Anaesthesiology, Super Speciality Hospital, Netaji Subhash Chandra Bose Medical College, Jabalpur, IND; 2 Anaesthesiology, All India Institute of Medical Sciences, Bhopal, Bhopal, IND; 3 Anaesthesiology, Sanjay Gandhi Postgraduate Institute of Medical Sciences, Lucknow, IND; 4 Microbiology, Bhopal Memorial Hospital and Research Centre, Bhopal, IND

**Keywords:** clonidine, dexmedetomidine, general anesthesia, laparoscopic cholecystectomy, nebulization, postoperative sore throat

## Abstract

Introduction

Postoperative sore throat (POST) is a common complication of endotracheal intubation. This study aims to compare the effect of ultrasonic nebulisation with dexmedetomidine versus clonidine on POST in patients undergoing elective laparoscopic cholecystectomy under general anaesthesia.

Methods

This prospective, observational, comparative cohort study was initiated after obtaining institutional ethics committee approval and registration with the Clinical Trials Registry of India (CTRI). In one group, 40 patients were ultrasonically nebulised with dexmedetomidine 50 mcg in 4 mL of saline, whereas in another group, 38 patients were ultrasonically nebulised with clonidine 100 mcg in 4 mL of saline. The primary outcome was to compare the efficacy of nebulised dexmedetomidine and clonidine in relieving POST, whereas the secondary outcome was to compare the effect of nebulised dexmedetomidine and clonidine on the haemodynamic parameters at defined time points.

Results

The demographic data of the study were comparable. The incidence of sore throat was less in the patients nebulised with dexmedetomidine compared to clonidine, which was more evident at six hours (p < 0.001), 12 hours (p < 0.001) and 24 hours after surgery (p = 0.027). The overall change in heart rate over time was comparable in the two groups. The overall change in mean arterial pressure over time was comparable in the two groups except at the time of intubation, when it was statistically significant (p = 0.032).

Conclusion

The study’s findings suggest that nebulisation with dexmedetomidine resulted in a lower incidence and less severe sore throat in postoperative patients compared to nebulisation with clonidine in patients undergoing elective laparoscopic cholecystectomy under general anaesthesia.

## Introduction

Postoperative sore throat (POST) is often described as throat discomfort (pain, scratchiness or irritation) that occurs during the postoperative recovery after surgery under general anaesthesia (GA) with airway instrumentation [1]. It often lasts up to 24 hours, but sometimes persists for up to 96 hours in some cases [2]. The incidence of POST varies from 18% to 65% in different studies [2-4]. In one study, tracheal intubation resulted in the highest POST incidence (45.4%) when compared to laryngeal mask airway (17.5%) and open mask (3.3%) [5].

POST hinders patients' ability to recover and interact with medical professionals. Patients may have difficulty swallowing as a result. In extreme situations, coughing and respiratory problems may result from a sore throat [1].

Many pharmacological and non-pharmacological methods have been suggested in the literature for attenuating POST with no proven single modality. In previous studies, nebulised forms of different drugs like ketamine, ketamine with clonidine, dexmedetomidine, dexamethasone, magnesium sulfate, budesonide and lignocaine have been used to decrease POST when administered in nebulised form in the preoperative period [6-11].

Dexmedetomidine and clonidine, both alpha-2 adrenergic agonists with primarily sympatholytic action, have not been compared for their effectiveness in reducing POST via preoperative ultrasonic nebulisation.

So, the primary objective of this study was to estimate and compare the efficacy of ultrasonic nebulisation with dexmedetomidine and clonidine on POST in patients undergoing elective laparoscopic cholecystectomy under GA with endotracheal intubation. The secondary objective was to estimate and compare the effect of ultrasonic nebulisation with dexmedetomidine and clonidine on perioperative haemodynamic parameters.

## Materials and methods

This prospective observational study was conducted in a tertiary care teaching hospital after approval from the institutional ethical committee (IHEC-PGR/2021/PG/Jul/03). This study was prospectively registered in the Clinical Trials Registry of India (CTRI/2022/04/042238). Written informed consent was taken from all the patients after a thorough explanation of the purpose and procedure of the study. Consenting patients aged 18-65 years, of either gender, with American Society of Anesthesiologists (ASA) physical status grades I or II, scheduled for elective laparoscopic cholecystectomy under GA with endotracheal intubation, were included in this study.

Patients with an allergy to study drugs, a difficult airway, multiple intubation attempts, active upper respiratory tract infections, preexisting sore throat or hoarseness, steroid or nonsteroidal anti-inflammatory drug use, sinus bradycardia, atrioventricular block, cardiac disease, chronic obstructive pulmonary disease, asthma, or a history of preoperative sore throat were excluded.

As this is a non-randomised study, patients were allocated into one of the study groups based on the anaesthesiologist's discretion. ASA standard fasting guidelines were followed in all study participants. Baseline readings of heart rate (HR), non-invasive blood pressure (NIBP) and peripheral oxygen saturation (SpO_2_) were noted in the preoperative area.

Dexmedetomidine is more potent than clonidine, and a previous study chose to compare 1 µg/kg (≈50 µg in adults) dexmedetomidine versus 2 µg/kg (≈100 µg) clonidine for attenuation of haemodynamic response to laryngoscopy and intubation [12]. As nebulised absorption is less predictable, considering pharmacological potency and prior safety data, we selected 50 µg dexmedetomidine and 100 µg clonidine as reasonably comparable and clinically relevant doses for nebulisation. Patients in the dexmedetomidine group (n = 40) were ultrasonically nebulised with 50 micrograms of dexmedetomidine (1 mL) and 3 mL of normal saline, whereas patients in the clonidine group (n = 38) were ultrasonically nebulised with 100 micrograms of clonidine (1 mL) and 3 mL of normal saline for 15 minutes before GA with endotracheal intubation.

Nebulisation was performed using an ultrasonic nebuliser (Sahyog Wellness Ultrasonic Nebuliser Machine (MY-520A), India) with an oxygen flow of 6 L/min for approximately 15 minutes, until the drug volume was completely exhausted. Standard anaesthesia monitors, including electrocardiogram, NIBP, and SpO_2_, were connected to the patient after transfer to the operating room.

In both groups, GA was induced with intravenous (IV) fentanyl (2 μg/kg), propofol (2 mg/kg) and vecuronium (0.1 mg/kg), followed by tracheal intubation with an appropriately sized endotracheal tube (ETT). Anaesthesia was maintained with IV fentanyl, vecuronium and isoflurane in an O_2_:N_2_O (40:60) to achieve 0.5-1 minimum alveolar concentration (MAC). Laryngoscopy and intubation were performed smoothly by the same experienced anaesthesiologist, lasting less than 15 seconds in all patients, to avoid interobserver bias. ETT intra-cuff pressure was monitored, and cuff overinflation was avoided. Normocapnia was maintained with controlled mechanical ventilation with end-tidal carbon dioxide (ETCO_2_) ranging from 30-35 mmHg. Measures were taken to maintain euvolaemia and normothermia. All patients received IV ondansetron (4 mg) 30 minutes before extubation in both groups. Gentle extubation of the patient was done after reversal of the neuromuscular blocking agent following completion of the surgery.

Haemodynamic parameters like HR and mean arterial pressure (MAP) were recorded at the following time points: baseline, induction, laryngoscopy, intubation, one minute after intubation, five minutes after intubation and after extubation. ETT cuff pressures were also noted after intubation, during gas insufflation of the abdomen and at extubation. In the postoperative period, sore throat was evaluated at 10 minutes, 1 hour, 6 hours, 12 hours and 24 hours after extubation. Sore throat severity was graded using the following classification: 0 - no sore throat; 1 - mild sore throat (complaints of sore throat on asking); 2 - moderate sore throat (complaints of sore throat on his/her own); and 3 - severe sore throat (change in voice or hoarseness associated with throat pain) [13]. Patients who developed a sore throat in any group were treated as per the institute protocol. Patients were encouraged to perform warm saline gargles and adequate hydration. Mild cases were managed with IV paracetamol and topical lignocaine spray, whereas moderate-to-severe POST was managed with nebulised dexamethasone.

Based on the previous study by Thomas et al. [1], to find a difference of 20.4% in the incidence of sore throat with an α error of 0.05 and power of 80%, the minimum sample size required in each group was 33. Assuming a 10% dropout, we decided to recruit 37 patients in each group for this study.

IBM SPSS Statistics for Windows, Version 23 (Released 2016; IBM Corp., Armonk, New York, United States) was used for data analysis. Descriptive statistics were elaborated as means/standard deviations and medians/interquartile ranges (IQRs) for continuous variables, and frequencies and percentages for categorical variables. Group comparisons for continuously distributed data were made using the independent sample t-test when comparing two groups, and one-way analysis of variance (ANOVA) when comparing more than two groups. Non-normally distributed data were analysed using the Wilcoxon test/Kruskal-Wallis test. Chi-squared test was used for group comparisons of categorical data. Statistical significance was kept at p < 0.05.

## Results

A total of 78 patients were enrolled over 18 months (dexmedetomidine, n = 40; clonidine, n = 38) for elective laparoscopic cholecystectomy. The demographic characteristics were comparable between the two groups (Table 1).

**Table 1:** Demographic information of participants. * Chi-squared; ** Wilcoxon-Mann-Whitney U test

Parameter	Groups		P-value
	Dexmedetomidine (n = 40)	Clonidine (n = 38)	
Age (years), mean ± standard deviation	42.80 ± 10.41	37.68 ± 9.24	0.390*
Gender, n (%)			
Male	24 (60.0)	17 (44.7)	0.177*
Female	16 (40.0)	21 (55.3)	
Duration of surgery (minutes), mean ± standard deviation	163.25 ± 36.89	156.32 ± 43.77	0.480**

The incidence of sore throat was lower in the dexmedetomidine group at all study time points; however, the difference was statistically significant at 6, 12 and 24 hours after extubation (Table 2).

**Table 2:** Sore throat incidence at various time intervals in both groups. * Fisher's exact test; ** Chi-squared test

Parameters	Groups		P-value
	Dexmedetomidine (n = 40)	Clonidine (n = 38)	
Sore throat (10 minutes), n (%)	0 (0.0)	1 (2.6)	0.487*
Sore throat (1 hour), n (%)	1 (2.5)	4 (10.5)	0.195*
Sore throat (6 hours), n (%)	5 (12.5)	19 (50.0)	<0.001**
Sore throat (12 hours), n (%)	5 (12.5)	18 (47.4)	<0.001**
Sore throat (24 hours), n (%)	1 (2.5)	7 (18.4)	0.027*

Sore throat grades at different time intervals were statistically significant between the two groups at 6, 12 and 24 hours after extubation (Table 3).

**Table 3:** Sore throat grades at various time intervals after extubation in both groups. * Fisher's exact test

Parameters	Groups		P-value
	Dexmedetomidine (n = 40)	Clonidine (n = 38)	
Sore throat grade (10 minutes), n (%)			
None	40 (100.0)	37 (97.4)	0.487*
Grade 1	0 (0.0)	0 (0.0)	
Grade 2	0 (0.0)	1 (2.6)	
Sore throat grade (1 hour), n (%)			
None	39 (97.5)	34 (89.5)	0.195*
Grade 1	1 (2.5)	4 (10.5)	
Grade 2	0 (0.0)	0 (0.0)	
Sore throat grade (6 hours), n (%)			
None	35 (87.5)	19 (50.0)	<0.001*
Grade 1	4 (10.0)	12 (31.6)	
Grade 2	1 (2.5)	7 (18.4)	
Sore throat grade (12 hours), n (%)			
None	35 (87.5)	19 (50.0)	<0.001*
Grade 1	4 (10.0)	12 (31.6)	
Grade 2	1 (2.5)	7 (18.4)	
Sore throat grade (24 hours), n (%)			
None	39 (97.5)	31 (81.6)	0.005*
Grade 1	0 (0.0)	7 (18.4)	
Grade 2	1 (2.5)	0 (0.0)	

The comparison of HR and MAP at different time points is presented in Tables 4-5, respectively.

**Table 4:** Comparison of heart rate at different point intervals in both groups. * Wilcoxon-Mann-Whitney U test; ** t-test

Parameters	Groups		P-value
	Dexmedetomidine (n = 40)	Clonidine (n = 38)	
Heart rate (baseline), mean ± standard deviation	81.88 ± 12.70	81.11 ± 14.41	0.406*
Heart rate (induction), mean ± standard deviation	82.28 ± 12.25	80.87 ± 9.26	0.638*
Heart rate (laryngoscopy), mean ± standard deviation	85.90 ± 10.25	84.84 ± 7.30	0.600**
Heart rate (intubation), mean ± standard deviation	87.10 ± 12.22	84.61 ± 7.88	0.285**
Heart rate (1 minute after intubation), mean ± standard deviation	80.03 ± 8.87	77.95 ± 9.26	0.315**
Heart rate (5 minutes after intubation), mean ± standard deviation	75.67 ± 10.53	75.11 ± 12.11	0.309*
Heart rate (after extubation), mean ± standard deviation	78.95 ± 14.81	77.95 ± 11.68	0.916*

**Table 5:** Comparison of mean arterial pressure at different point intervals in both groups. * t-test

Parameters	Groups		P-value*
	Dexmedetomidine (n = 40)	Clonidine (n = 38)	
Mean arterial pressure (mmHg) (baseline), mean ± standard deviation	98.58 ± 10.41	92.37 ± 9.99	0.09
Mean arterial pressure (mmHg) (induction), mean ± standard deviation	89.10 ± 12.66	85.53 ± 12.48	0.213
Mean arterial pressure (mmHg) (laryngoscopy), mean ± standard deviation	89.55 ± 13.73	89.82 ± 14.87	0.935
Mean arterial pressure (mmHg) (intubation), mean ± standard deviation	86.28 ± 16.98	96.13 ± 18.98	0.032
Mean arterial pressure (mmHg) (1 minute after intubation), mean ± standard deviation	79.12 ± 11.12	80.47 ± 10.98	0.738
Mean arterial pressure (mmHg) (5 minutes after intubation), mean ± standard deviation	84.00 ± 12.31	79.84 ± 9.61	0.100
Mean arterial pressure (mmHg) (after extubation), mean ± standard deviation	88.15 ± 11.07	86.32 ± 11.02	0.466

ETT cuff pressures at the time of intubation (p = 0.104), during abdominal gas insufflation (p = 0.860), and at extubation (p = 0.208) were comparable between the two groups. As patients with difficult airways were excluded, neither a stylet nor a bougie was used for intubation; all patients were intubated using direct laryngoscopy. None of the patients had sleep apnoea. As per institutional protocol, a nasogastric tube was inserted in all patients following endotracheal intubation. No patient had a history of hiatus hernia or heartburn. Bag-mask ventilation was not difficult, and no additional instrumentation was performed in the oral cavity.

## Discussion

This study was intended to compare the effect of ultrasonic nebulisation with dexmedetomidine and clonidine on POST in patients undergoing elective laparoscopic cholecystectomy under GA with an ETT. The data suggest that dexmedetomidine results in a lower incidence of sore throat compared to clonidine at various time intervals, with statistically significant differences observed at 6, 12, and 24 hours. Sore-throat severity was greater in the clonidine group than in the dexmedetomidine group.

The overall incidence of POST in our present study was 1.2% at 10 minutes, 6.4% at 1 hour, 30% at 6 hours, 29% at 12 hours and 10% at 24 hours. It was similar to earlier studies, which reported POST in the range of 18-74% [2,3,14,15]. The lesser incidence and lesser severity of POST in the dexmedetomidine group in comparison to the clonidine group may be supported by multifaceted protective functions of dexmedetomidine. Dexmedetomidine selectively activates α2-adrenaline receptors in the locus coeruleus and is a derivative of medetomidine, which inhibits the sympathetic nervous system and reduces the release of norepinephrine [16]. Dexmedetomidine has been proven to reduce the pro-inflammatory cytokine production, attenuate the inflammatory response and inhibit pain signals [17-20].

IV dexmedetomidine was found to significantly reduce the incidence of POST in a meta-analysis [21]. However, in our study, we used dexmedetomidine through nebulisation and found similar results. These results were further supported by a study where the authors concluded that pharmacological effects were similar with both intranasal and IV routes of administration [22].

In a study where dexmedetomidine was compared with ketamine through the nebulisation route, the authors opined that dexmedetomidine nebulisation is a safe method in attenuating POST, with fewer haemodynamic derangements [1]. These results were in correlation with our patients in the dexmedetomidine group. However, the authors from a recent study found that preoperative dexmedetomidine nebulisation does not affect the POST, although the intraoperative anaesthetic and analgesic consumption was found to be less [23].

There is only sparse literature available regarding the use of preoperative clonidine nebulisation on the effect on POST. In one study, where ketamine nebulisation was compared with ketamine and clonidine nebulisation, the results showed that preoperative ketamine plus clonidine nebulisation was more effective in reducing POST with no adverse effects [11].

We found that there was no significant difference in the trend of HR at any of the time points in both groups. The overall change in MAP over time was comparable in the two groups except at the time of intubation, when it was statistically significant (p = 0.032). The fine mist generated by ultrasonic nebulisers allows a high proportion of drug to reach the target airway sites, potentially increasing therapeutic efficacy [24].

Various studies have investigated the comparative effects of IV dexmedetomidine versus IV clonidine on the haemodynamic response to laryngoscopy and intubation. In these studies, they found that dexmedetomidine consistently demonstrates superior efficacy in attenuating increases in HR and MAP during laryngoscopy, intubation and intraoperative periods compared to clonidine [15,25].

Our results differ from their results, probably due to the administration of dexmedetomidine through different routes, hence affects their bioavailability. Increased ETT intracuff pressure is suggested as one of the risk factors for POST [15]. In this study, ETT intracuff pressure is comparable in the dexmedetomidine and clonidine groups.

The strength of this study is its novelty, as we could not find any study comparing the effect of ultrasonically nebulised dexmedetomidine and clonidine on POST. The present study has certain limitations too. Firstly, this is a non-randomised study, so the chance of bias could not be ruled out completely. Secondly, we recruited only ASA I and ASA II patients, so our results could not be extrapolated uniformly to high-risk patients. Thirdly, we didn’t measure the plasma concentrations of study drugs, and no formal sedation scale was used. Finally, we could not rule out the contribution of the systemic effects of the study drugs. In both groups, intraoperative analgesia was standardised using IV fentanyl and IV paracetamol to minimise potential confounding effects. Only ASA I and ASA II patients were enrolled, as specified in the methodology, and none of the participants had systemic comorbidities, thereby excluding comorbidities as potential confounders. The duration of surgery was comparable between the two groups, with no statistically significant difference observed (p = 0.480), thus ruling out surgical duration as a confounding factor. Although we considered all the potential confounders, adjusting for these confounders using an appropriate statistical test would isolate the specific effects of nebulised dexmedetomidine and clonidine on POST, improving the precision and reliability of the findings. Patients were not monitored every five minutes after the administration of the study drugs, and CO_2_ monitoring was not performed during preoperative administration of the test drugs; these were additional limitations of the study.

## Conclusions

In this observational study, preoperative ultrasonic nebulisation with dexmedetomidine was associated with a lower incidence and severity of POST compared with clonidine in patients undergoing elective laparoscopic cholecystectomy under GA with endotracheal intubation. However, given the study’s observational design, the findings reflect an association rather than causation, and potential biases and unmeasured variables may have influenced outcomes. Well-designed multicenter randomised controlled trials with larger sample sizes are warranted to confirm these preliminary observations.
